# An Exploration of Hypotheses that Explain Herbivore and Pathogen Attack in Restored Plant Communities

**DOI:** 10.1371/journal.pone.0116650

**Published:** 2015-02-20

**Authors:** G. Kai Blaisdell, Bitty A. Roy, Laurel Pfeifer-Meister, Scott D. Bridgham

**Affiliations:** Institute of Ecology and Evolution, Department of Biology, University of Oregon, Eugene, Oregon, United States of America; Oklahoma State University, UNITED STATES

## Abstract

Many hypotheses address the associations of plant community composition with natural enemies, including: (i) plant species diversity may reduce enemy attack, (ii) attack may increase as host abundance increases, (iii) enemy spillover may lead to increased attack on one host species due to transmission from another host species, or enemy dilution may lead to reduced attack on a host that would otherwise have more attack, (iv) physical characteristics of the plant community may influence attack, and (v) plant vigor may affect attack. Restoration experiments with replicated plant communities provide an exceptional opportunity to explore these hypotheses. To explore the relative predictive strengths of these related hypotheses and to investigate the potential effect of several restoration site preparation techniques, we surveyed arthropod herbivore and fungal pathogen attack on the six most common native plant species in a restoration experiment. Multi-model inference revealed a weak but consistent negative correlation with pathogen attack and host diversity across the plant community, and no correlation between herbivory and host diversity. Our analyses also revealed host species-specific relationships between attack and abundance of the target host species, other native plant species, introduced plant species, and physical community characteristics. We found no relationship between enemy attack and plant vigor. We found minimal differences in plant community composition among several diverse site preparation techniques, and limited effects of site preparation techniques on attack. The strongest associations of community characteristics with attack varied among plant species with no community-wide patterns, suggesting that no single hypothesis successfully predicts the dominant community-wide trends in enemy attack.

## Introduction

Incorporating ecological theory concerning natural enemies (herbivores and pathogens) into native plant conservation efforts is problematic, because many competing hypotheses address associations between host community composition and natural enemies, with varying levels of empirical support. Well-replicated plant community restoration studies are an exceptional opportunity to explore existing hypotheses concerning plant community dynamics, providing plant communities with similar seed rain and replication of taxa among experimental communities. In this study, we used a well-replicated native plant community restoration experiment that tested several initial site preparation techniques, to (i) explore the relative strength of multiple hypotheses to successfully explain host-natural enemy dynamics across the plant community, and (ii) learn how natural enemy attack on plants varied among the site preparation treatments, presumably due to variation among plant communities. In the following paragraphs, we discuss merits and possible shortcomings of several often-cited hypotheses that relate to plant community structure and natural enemy attack. Then we describe how we explored each of these hypotheses.

One host-enemy community pattern that has been presented in primary literature is that enemy attack tends to decrease as host diversity increases [1–4]. Yet the relationship between host diversity and enemy attack is not always negative or simple [5–8]. Studies suggest that the strength of support for this pattern is dependent on the methods used to examine it. For example, some studies examined diversity effects on the attack of a single focal host species, ignoring community-wide trends of enemy attack [9–11]. However, a decrease in enemy attack as host richness increases is most strongly supported at low species richness levels, with ambiguous results at higher species richness [2].

Low host abundance, measured as density or frequency, may limit natural enemies due to difficulty of dispersal among suitable hosts [12–14]. Hypotheses relating to host abundance and herbivore attack generally predict increased attack as host abundance increases [15]. Many studies have focused on abundance of one particular host species and enemy attack on that species, with mixed findings; herbivory can either decrease or increase with increased host availability, and can be affected by how closely related plant species within the community are [9,10,16]. Few studies, however, have examined community-wide patterns of abundance of multiple hosts and enemy attack on each of those host species; an analysis of multiple dominant host species in a community may reveal different host abundance-natural enemy relationships among host species, or a common trend among host species in patterns describing the dynamics between natural enemy attack and host abundance. Additionally, studies and discussion of host communities often fail to distinguish between the effects of host diversity and host abundance. For example, Keesing and others [4] indicated that the “diversity effect” found by Mitchell and others [2] was actually an artifact of the density of one particular host species and a specialist pathogen that attacked only that host. Careful consideration is needed to separate the effects of host species diversity from abundance of individual host species.

As a particular natural enemy population increases on its favored host species, the natural enemy may then spill over onto neighboring species, which would suffer more attack than in the absence of the favored host species [17]. Conversely, as the favored host species becomes less common, the neighboring species can dilute the natural enemy in the community. Dilution and spillover can be affected by the identity of one or more host species in a community, and are therefore distinct from a more general effect of host species diversity or host abundance.

Enemy spillover from introduced to native plant species has been implicated in facilitating invasions in plant communities [18–21], which may be of concern in native plant community restoration efforts. Parker et al. [22] showed in a meta-analysis that native herbivores actually suppress introduced species, while introduced herbivores lead to a proliferation of their introduced hosts. Furthermore, these relationships are stronger when the hosts are more closely related [23]. These observed broad patterns may be due to spillover and dilution dynamics between introduced and native plant species. More information is needed to consider community-wide patterns of natural enemy spillover and dilution among individual host species, as well as possible spillover and dilution dynamics that may influence the success of invasive plant species.

The physical structure of the plant community can affect populations of herbivores and pathogens. Total vegetation cover affects microclimate, including light, temperature, and humidity. High humidity in dense stands usually favors infection and sporulation, but can impede dispersal of newly formed inoculum [24,25]. Sun and shade have species-specific effects on enemies [26,27]. A buildup of dead plant material (thatch) can harbor pathogenic fungi. For example, in our study area in Oregon, USA, farmers use field burning after harvest to reduce crop residue and pathogens harbored there [28].

The plant vigor hypothesis [29] predicts that larger, more vigorous plants will experience more attack by herbivores than stressed plants, because larger plants are a more desirable target for natural enemies [14,30]. Higher nitrogen content per leaf area has been found to increase foliar fungal disease [31,32], and the carrying capacity and population growth rate of aphids [33]. Alternatively, the plant stress hypothesis predicts that stressed plants will experience more herbivore attack [34]. Two meta-analyses of herbivore studies have found support for the plant vigor hypothesis, but not for the plant stress hypothesis [35,36].

Patterns of natural enemy attack in plant community restoration, which could affect the success of rehabilitation efforts, have not been extensively studied, and that was a goal of our project. Our study site was a seasonal wetland prairie restoration experiment in Eugene, Oregon, USA [37,38], that was designed to test the effects of various site preparation techniques on native plant community restoration. The restoration experiment provided an ideal setting for examining interactions between plant community characteristics and natural enemy attack, because it created different plant communities with some shared species in an area that has a relatively homogeneous physical environment.

Our study had two objectives: to (1) compare the relative strengths of several hypotheses in their ability to explain community-wide patterns of natural enemy attack, and (2) compare the response of community-wide natural enemy (foliar herbivore and fungal pathogen) attack among site preparation techniques. We examined variation of natural enemy attack on six native grass and forb species, among ten site preparation techniques and a nearby extant wetland prairie. Detailed plant cover data enabled us to address several patterns of plant community effects on natural enemies, including the effects of diversity, relative abundance of introduced species, relative abundance of affected species, physical attributes of the community structure, and individual plant traits on enemy attack. Our survey included six native plant species, which combined comprised 80% of total plant cover, in communities ranging in species richness from 8 to 26 plant species per m^2^. We include in our analyses separate measures of relative abundance of each host species surveyed and plant species diversity. We looked for correlations of enemy attack on each plant species with the relative abundance of several plant species, as such correlations would likely be due to spillover or dilution. We also considered possible spillover effects from introduced to native species. We analyzed variation in natural enemy attack relative to physical characteristics of the plant community, including total plant cover and abundance of thatch. To explore the plant vigor and plant stress hypotheses, we measured nutrient content and aboveground biomass of each plant sampled as estimates of plant vigor, and analyzed variation in natural enemy attack relative to these individual plant traits. We hypothesized that (i) plant species diversity would be negatively correlated with natural enemy attack on plants, (ii) enemy attack would increase as host abundance increased, (iii) the relative abundance of individual plant species would either be positively or negatively correlated with enemy attack on other plant species, respectively suggestive of spillover or dilution, (iv) physical traits of the plant community, such as total plant cover and thatch, would be positively correlated with natural enemy attack, and (v) traits of individual plants, such as size or nutrient content, would be positively correlated with attack on those plants as predicted by the plant vigor hypothesis, or negatively correlated as predicted by the plant stress hypothesis.

## Materials and Methods

### Study site

Our survey was performed in a restoration experiment, established in 2004, that was designed to test how site preparation techniques affect the relative success of native and introduced plant species [37,38]. Prior to the restoration experiment, the area had been planted in *Lolium multiflorum* Lam. (annual ryegrass, Poaceae) for grass seed production. The 4.5 ha experiment included ten experimental land preparation techniques, replicated five times using randomized 15 m^2^ plots, for a total of 50 plots. Plots were separated by 10 m mowed buffers, and a 23 m mowed buffer surrounded the experimental restoration site. The treatments included ten combinations of tilling, herbicide application, thermal application, and solarization: till only, herbicide only, herbicide + thermal, two herbicide applications, till + herbicide, till + two herbicide applications, till + solarization, till + thermal, till + herbicide + solarization, and till + herbicide + thermal. Application of all treatments and broadcast seeding of 15 native grass and forb species were completed in October 2004, and natural succession was allowed to occur. We also surveyed a nearby remnant wetland prairie, using the same methods of measuring plant cover and individual plant traits as in the restoration experiment, for comparison to the restoration treatments. This reference wetland prairie was 4 km from the restoration experiment, and had the same soil type and similar hydrology relative to the restoration experiment [37,38].

### Description of site preparation techniques

Tilling can reduce the introduced seed bank in recently cultivated fields. As a result of tilling, seeds present in the soil are moved to the surface and germinate, thus reducing future germination of introduced species [39]. Thermal treatments are applied using infrared burners, which produce temperatures ranging from 540–1090° C. Plants’ cells are ruptured due to the resulting heat exposure. Thermal treatments may provide a desirable alternative to burning, without the undesirable things that accompany burning, such as excessive smoke and a risk of fire accidentally spreading from controlled burn sites. Solarization is the generation of heat and humidity over an extended time period by covering a large area of the ground with plastic. Solarization causes seeds in the seed bank to germinate and then die, thus reducing future germination [39]. Herbicide application reduces existing vegetation. In this restoration experiment, the herbicide applied was the broad-spectrum glyphosate. Practitioners and researchers have found that combining multiple site preparation techniques that reduce both the introduced seeds and extant vegetation at different stages seems to improve results [39].

### Collection of data

We collected plant cover data in June 2006, at peak standing biomass, using the point-intercept method [40] in one randomly located 1 m^2^ subplot per 15 m^2^ plot, for a total of 5 replicates per treatment. In the reference wetland prairie, five 15 m^2^ plots were randomly chosen, within which one 1 m^2^ subplot was randomly located in each 15 m ^2^ plot. Percent cover was recorded for each species using a 1 m^2^ frame with 25 pins. Species that were present in the 1 m^2^ subplot but did not contact a pin were allocated 1% cover. Additionally, dead plant material was recorded as present or absent for each pin, with each pin hit counting as 4% cover. Standing thatch was defined as dead plant material that was still standing, and ground thatch was defined as dead plant material lying horizontally on the ground. In 2006, the mean number of plant species richness per plot was 14, and the range was 8–26 species per plot. Plant diversity was calculated using Simpson’s diversity index based on the cover data collected. Relative abundances of each native host species, all introduced species, and the introduced *Lolium multiflorum* were calculated from the cover data. Total cover and percent cover of thatch were used to describe physical characteristics of the plant community.

To examine how the restoration treatments and plant community structure affected enemy attack on the native plants in the site, we assessed natural enemy damage on the six most common native perennial plant species, *Agrostis exarata* Trin. (spike bent grass, Poaceae), *Deschampsia cespitosa* (L.) P. Beauv. (tufted hair grass, Poaceae), *Madia glomerata* Hook. (tarweed, Asteraceae), *Prunella vulgaris* L. (common selfheal, Lamiaceae), *Epilobium densiflorum* (Lindl.) Hoch & P. H. Raven (willow herb, Onagraceae), and *Grindelia integrifolia* DC (gumweed, Asteraceae), in all ten restoration treatments and the reference wetland prairie.

All six native species had been seeded in all ten restoration treatments. These species combined comprised 80% of total plant cover in the study (Table 1). Plant phenology can affect susceptibility to particular guilds of natural enemies, and for this reason we sampled all plant species based on their phenological stage [41,42]. The survey spanned from June through August 2006, after flowering but before mature seed set or substantial senescence for each species. To associate detailed information about plant community composition and cover with enemy attack, natural enemy attack was surveyed on one plant of each native species, collected from within 10 cm of the subplot used to measure cover in each plot. This allowed us to associate natural enemy attack with detailed plant community data in each plant’s immediate environs.

**Table 1:** Community Factors that Were Measured and Entered into AIC Analysis.

Species or Factor	Mean	Standard Error	Minimum	Maximum	n
*Agrostis exarata*	41.0%	3.29	<0.1%	88.2%	55
*Deschampsia cespitosa*	23.8%	2.75	0.2%	95.0%	55
*Madia glomerata*	7.0%	1.09	0.0%	30.0%	53
*Prunella vulguaris*	5.9%	0.87	<0.1%	27.0%	55
*Epilobium densiflorum*	1.8%	0.42	0.0%	13.9%	39
*Grindelia integrifolia*	0.3%	0.07	0.0%	2.2%	32
**Total**	**80.2%**				**286**
*Lolium multiflorum*	5.5%	1.15	0.0%	46.3%	
Introduced species	14.6%	2.26	0.1%	65.4%	
Total cover	790%	25.41	422%	1389%	
Standing Thatch	85%	1.82	40%	100%	
Ground Thatch	43%	2.60	8%	96%	
Diversity (Simpson’s)	0.63	0.02	0.10	0.91	

Values indicate the average relative abundance of each species sampled. Total cover was measured in three dimensions, with no upward bound. Thatch was measured in two dimensions, bound at 100%. Means, standard errors, and the range (minimum and maximum) are reported across all plots, with n representing the number of plots in which each species was found and surveyed for herbivore and pathogen damage. For each species, only the plots in which that species was found are included in the statistical analyses for all factors considered. The first six plant species listed were surveyed for foliar herbivore and pathogen damage, and are therefore grouped separately from the other community factors in this table.

Percent visible foliar arthropod herbivore and fungal pathogen damage were assessed on each of three leaves from each plant, and the average of the three leaves was used. Percent damage was scored as a continuous variable. Percent damage caused by each type of symptom on each leaf was also scored; herbivore attack was scored as chew, rasp, mine, or sucking damage. Pathogen attack was scored as blotch, spot, or rust pustules (Table 2).

**Table 2:** Natural Enemy Guilds.

Plant Species	Chew	Rasp	Mine	Suck	Blotchy Pathogens	Spot Pathogens	Rust Pustules
*Agrostis exarata*	7.4	5.6	1.9	68.5	3.7	3.7	83.3
*Deschampsia cespitosa*	7.2	3.6	0	41.8	0	80.0	3.6
*Epilobium densiflorum*	66.7	35.9	0	7.7	10.2	35.9	18.0
*Grindelia integrifolia*	96.9	31.3	12.5	3.1	46.9	18.8	0
*Madia glomerata*	20.4	14.8	0	1.9	11.1	0	50.0
*Prunella vulgaris*	28.3	49.1	13.2	0	20.8	94.3	3.8

Types of arthropod herbivore and fungal pathogen symptoms found on plant species surveyed, and percent of plants surveyed on which each symptom type was found.

We used aboveground biomass and leaf nitrogen content of each plant as estimates of plant vigor. Aboveground biomass was measured at the time of sampling by clipping and drying at 60°C for at least 48 hours before weighing (Table 3). Leaf nitrogen content was estimated by measuring chlorophyll content in the field using a hand held portable SPAD-502 chlorophyll meter (Spectrum Technologies, Inc., Plainfield, Illinois, USA). For each plant, one measurement was taken for each of three leaves, and the average of the three measurements was used (Table 3). Damaged sections of leaves were avoided during chlorophyll measurements. Within plant species, chlorophyll content is highly correlated with nitrogen content [43,44]. A SPAD 502 meter can be used for quick, non-destructive and inexpensive estimation of nitrogen content [45].

**Table 3:** Individual Plant Variables.

Species	Biomass (grams)					Chlorophyll Content (SPAD Units)			
	N	Mean	SE	Min	Max	Mean	SE	Min	Max
*Agrostis exarata*	55	4.21	0.88	0.01	36.6	11.3	0.94	0.37	33.8
*Deschampsia cespitosa*	55	4.34	1.25	0.02	58.8	6.1	0.44	1.3	16.4
*Madia glomerata*	53	0.12	0.02	0.01	1.2	6.0	0.55	0.80	20.5
*Prunella vulgaris*	55	0.84	0.18	0.01	6.4	29.5	0.65	20.0	40.8
*Epilobium densiflorum*	39	0.08	0.02	0.004	0.6	13.1	1.03	1.6	28.2
*Grindelia integrifolia*	32	0.70	0.21	0.02	6.6	18.9	0.61	11.4	26.6

Chlorophyll content and above-ground shoot biomass were measured and entered into AIC analysis to explore the plant vigor and plant stress hypotheses. Number of individual plants of each species is represented by (n), and corresponds to the number of plots in which each plant species was present. Mean and standard error (SE) represent the mean value and standard error among all plants of each species in the survey. The minimum (Min) and maximum (Max) represent the plant with the lowest and highest values for each variable.

All sampling was performed on property owned by the City of Eugene, Oregon, with permission granted by the City of Eugene Department of Parks and Open Space, and was in compliance with all relevant laws and regulations.

### Data analysis

Analyses and results of the variation in natural enemy attack among restoration treatments are reported in (S1 File). Our examination of natural enemy attack in the different restoration treatments was partially motivated by our observation of large plant community differences among the restoration treatments during the previous growing season, in 2005. However, the differences in plant community composition among the restoration treatments dampened over time, and the plant communities in the restoration treatments became more similar to the composition of the reference prairie in 2006, the year in which our survey was conducted [37,38]. Even though there were not clear differences in plant community composition among the ten treatments and reference site, there were community differences among the 55 plots (Table 1), and the lack of treatment effects allowed us to more directly address how herbivore and pathogen attack varied with plant community composition and other factors. We tested the variation in vegetation characteristics among the 55 plots against several hypotheses whose goal is to explain dynamics between plant community composition and natural enemy attack.

To explore several hypotheses concerning what drives patterns in herbivore and pathogen attack in plant communities, we used Akaike’s Information Criterion (AIC) with multi-model inference [46] to check all possible subset models from the 14 predictor variables, and to select groups of equivalent models that would best explain herbivore and pathogen attack on each of the six native species [47]. The 14 predictor variables are individual shoot biomass, individual plant chlorophyll content (Table 3), and the 12 community variables listed in the first column of Table 1. When several potential models, i.e. combinations of predictor variables, are equivalent in that they have similar explanatory power, multi-model inference allows one to consider all equivalent models, rather than relying on the arbitrary selection that can occur with stepwise model selection [46–48]. The models tested included all possible combinations of the following 14 variables- community measures: Simpson’s diversity index (to explore diversity vs. enemy attack), relative abundance of each of the six native species sampled (to explore host abundance, spillover, and dilution), relative abundance of the introduced *Lolium multiflorum* (to explore spillover from *L*. *multiflorum*), relative abundance of introduced species (to explore spillover from introduced species), and total plant cover, percent ground thatch, and percent standing thatch (to explore physical characteristics of the plant community), (Table 1), and the following individual plant measures: aboveground biomass and chlorophyll content (to explore the plant vigor and plant stress hypotheses) (Table 3). We used Simpson’s diversity index because of its high sensitivity to species evenness [49]. All subset models were tested. We selected all models for which the corrected AIC value differed from the minimum corrected AIC value by less than two [46]. Using corrected AIC values provided a correction for relatively small and different sample sizes among plant species (n = 32–55, Table 1). We explored each host species and enemy type (herbivore or pathogen) separately, to determine the best set of predictors of herbivore and pathogen attack on each of the six native plant species surveyed. Because some plant species surveyed did not occur in all plots sampled, the values of the community factors varied somewhat among the six species. All analyses were performed using JMP version 7.0.1.

Each of the 14 predictor variables entered into our AIC analyses and multi-model inference was selected a priori and addressed a specific hypothesis. Some variables showed pair-wise correlations and variance inflation factors high enough to raise concern [50,51]. For example, in our worst case scenario, the relative abundance of introduced species with the relative abundance of *L*. *multiflorum* had a partial correlation of 0.87 for the analysis of *E*. *densiflorum*. When pair-wise correlations were worrisome, and removing one variable could potentially hurt our analysis in other ways [52], to assure model stability we used stepwise regression of the variables that were selected in our multiple models that were selected by our corrected AIC analyses, with both variables and each variable excluded (data not shown). Furthermore, others have shown that AIC multi-model inference is robust against error with variable pair-wise correlations up to 0.94 [46,53]. Another potential pitfall is that the relative abundances of *L*. *multiflorum* and introduced species contain overlapping information because *L*. *multiflorum* was the most common introduced species in many plots. However, in a previous survey of the three most common grasses in a subset of the restoration treatments, we found that the introduced *L*. *multiflorum* had more pathogen damage than the two native grasses *A*. *exarata* and *D*. *cespitosa* [54]. Based on these results we hypothesized that the pathogen may spread from the *L*. *multiflorum* to the two native grasses, and we decided that the benefit of keeping both *L*. *multiflorum* and introduced species abundance in the analysis outweighed the costs of potential bias. Others have included variables that contain overlapping information using AIC [55]. The strength of our analyses is the number and breadth of hypotheses addressed regarding the effects of community variables on natural enemy attack across the dominant species in the community.

## Results

**Fig 1:**
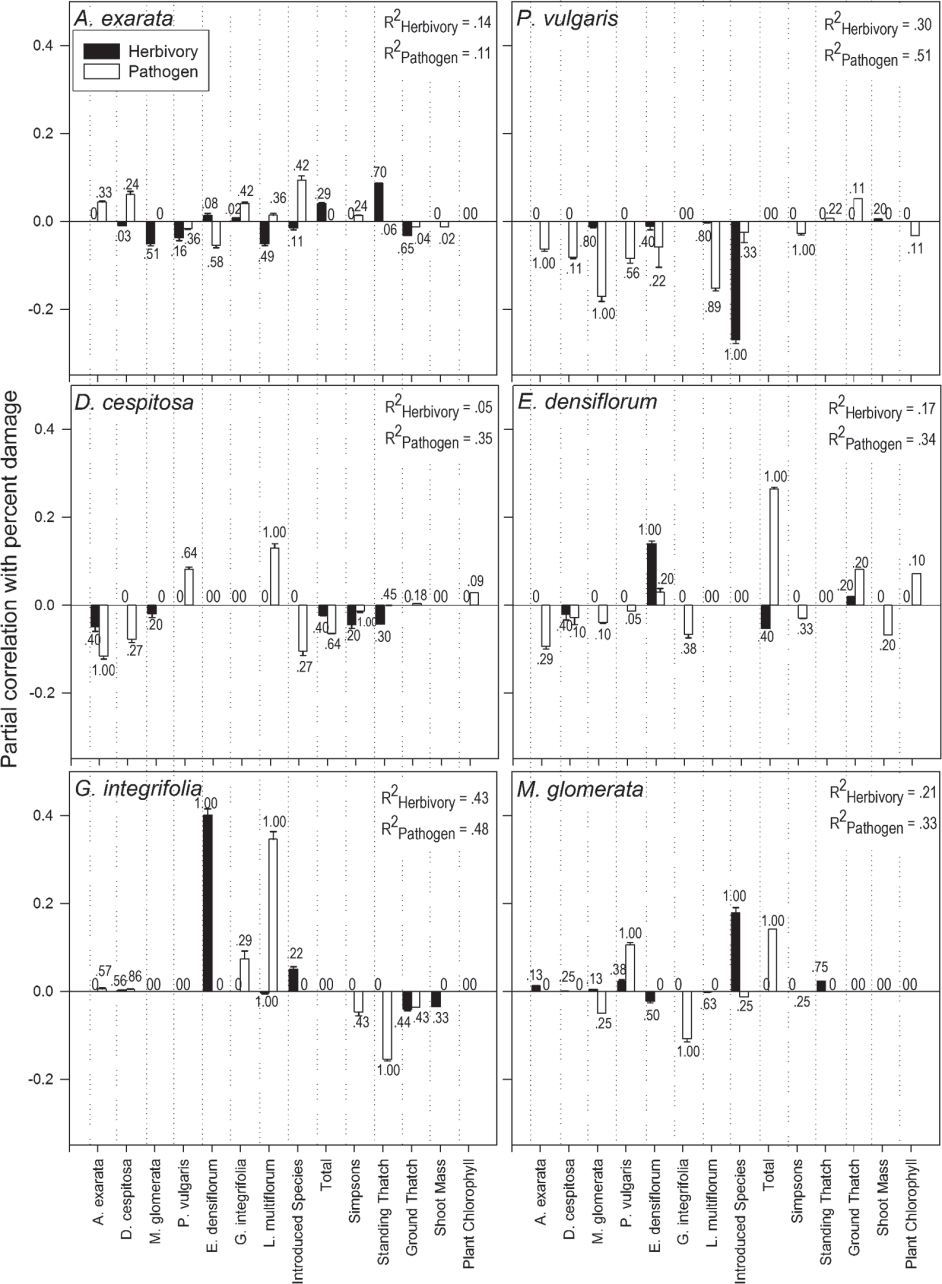
Average Partial Correlations from Multiple Regression Models of 14 Predictor Variables Regressed against Herbivore/Pathogen Attack. Error bars (standard error) are shown among partial correlations of each variable across all models in which that variable was selected. Numbers above each bar represent the proportion of models in which that variable was selected. Error bars represent variation in magnitude of partial correlation among selected models. R^2^ values represent the average predictive power of the multiple selected models for each host species-natural enemy combination. Figs. 2 and 3 depict subsets of the data shown in this figure, arranged according to hypothesis rather than host species. Predictor variables are defined as follows; the first seven predictor variables represent relative abundance of each species denoted as percent of total plant cover, Introduced Species: relative abundance of introduced plant species as percent of total plant cover, Total: total plant cover (see manuscript for method of recording total plant cover), Simpsons: Simpson’s diversity index, Standing Thatch and Ground Thatch: percent cover of thatch, Shoot Mass: above-ground individual shoot biomass, Plant Chlorophyll: leaf chlorophyll content.

Multiple regression analysis using AIC on 14 potential explanatory factors revealed that different factors influenced herbivores versus pathogens, and the importance of individual factors differed among the six native plant species (Fig. 1). In general, the models selected explained more of the variation in pathogen damage than herbivore damage. Mean model explanatory power for herbivory ranged from R^2^ = 0.05 (*Deschampsia cespitosa*) to R^2^ = 0.43 (*Grindelia integrifolia*). Mean model explanatory power for pathogen attack ranged from R^2^ = 0.11 (*Agrostis exarata*) to R^2^ = 0.51 (*Prunella vulgaris*) (Fig. 1). Individual predictor variables ranged as high as R^2^ = 0.40 (herbivory on *G*. *integrifolia* as relative abundance of *Epilobium densiflorum* increased).

**Fig 2:**
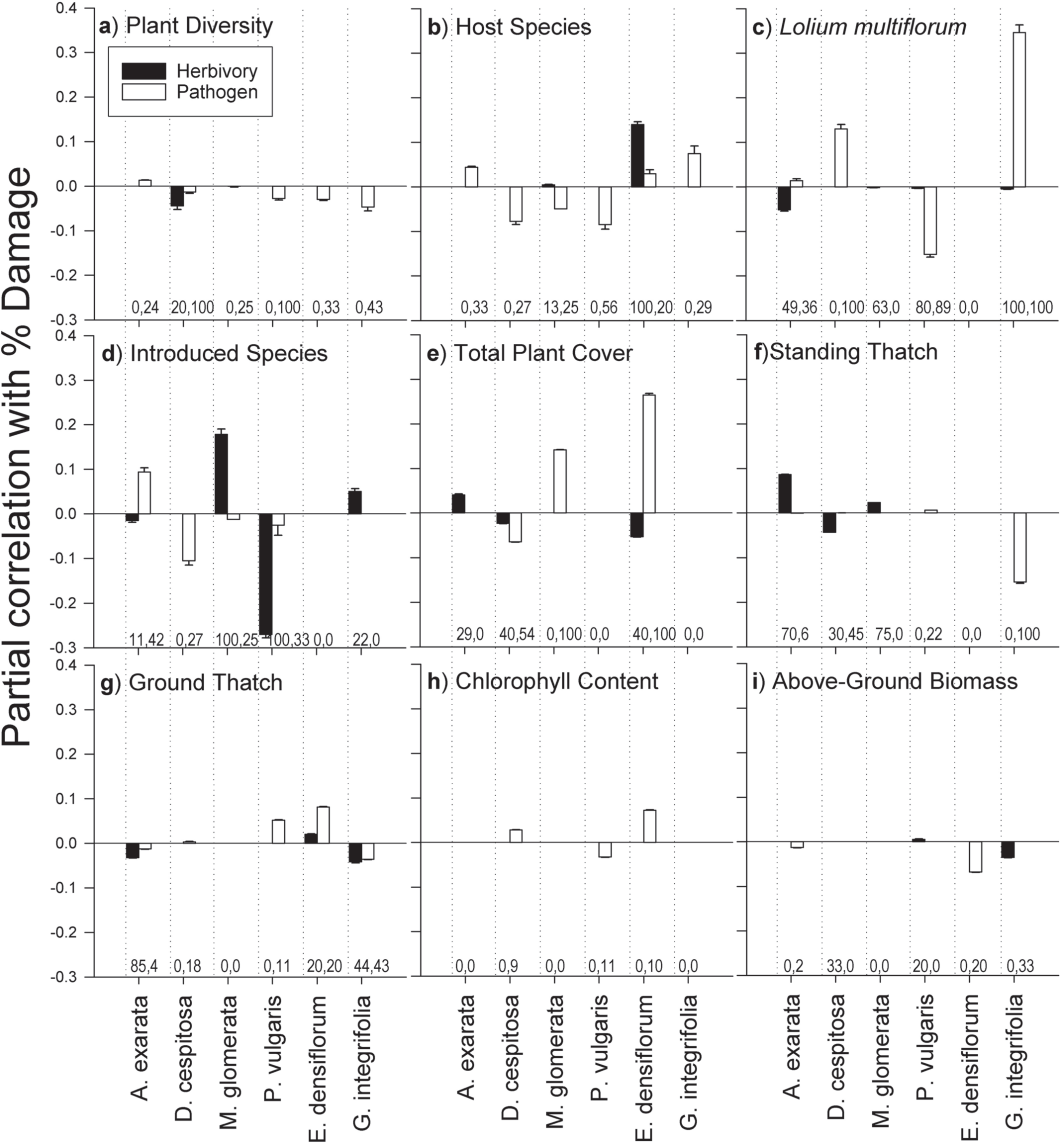
Partial Correlations of Variables with Herbivore and Pathogen Attack to Six Native Species. Title of each panel is the variable of interest for that panel. Panels **b**, **c**, and **d** represent relative abundance of the variable of interest. Panels **f** and **g** represent percent cover of the variable of interest. Mean partial correlations with the variable of interest and percent herbivore or pathogen attack on each of six species are represented by bars. Error bars represent variation (standard error) in magnitude of partial correlation among selected models. Numbers along x axes below each bar represent the percent of models in which that variable was selected (herbivory, pathogen).

As plant species diversity increased, there were no large increases or decreases in enemy attack on any one of the six plant species (Fig. 2A). There was no strong or consistent relationship between herbivore attack and plant species diversity. There was, however, a slight negative correlation with plant species diversity and pathogen attack on five of the six species: *D*. *cespitosa*, *Madia glomerata*, *P*. *vulgaris*, *E*. *densiflorum*, and *G*. *integrifolia*. This relationship, though small, appeared in all selected models for two of the plant species, *D*. *cespitosa* and *P*. *vulgaris*.

We found a correlation between host plant species abundance and enemy attack on that host plant species for only one plant species; as *E*. *densiflorum’*s relative abundance increased, herbivore attack on *E*. *densiflorum* also increased, and this predictor variable appeared in all models selected (Fig. 2B). We found no relationships between host plant species abundance and pathogen attack on *E*. *densiflorum* or host plant species abundance and enemy attack on the other five plant species.

As relative abundance of the most common introduced plant species *Lolium multiflorum* increased, pathogen attack on the native grass *D*. *cespitosa* and the native forb *G*. *integrifolia* increased (Fig. 2C). Relative abundance of *L*. *multiflorum* appeared in all selected models for these two species. In contrast, as relative abundance of *L*. *multiflorum* increased, pathogen attack on *P*. *vulgaris* decreased. *Lolium multiflorum* appeared in 89% of the models selected for *P*. *vulgaris*. Other partial correlations for *L*. *multiflorum* were very small or appeared in a small fraction of the selected models. As relative abundance of introduced plant species increased, herbivore attack decreased on *P*. *vulgaris* but increased on *M*. *glomerata* (Fig. 2D). Overall, correlations of natural enemy attack with abundance of introduced species were host species-specific, following no community-wide trends.

**Fig 3:**
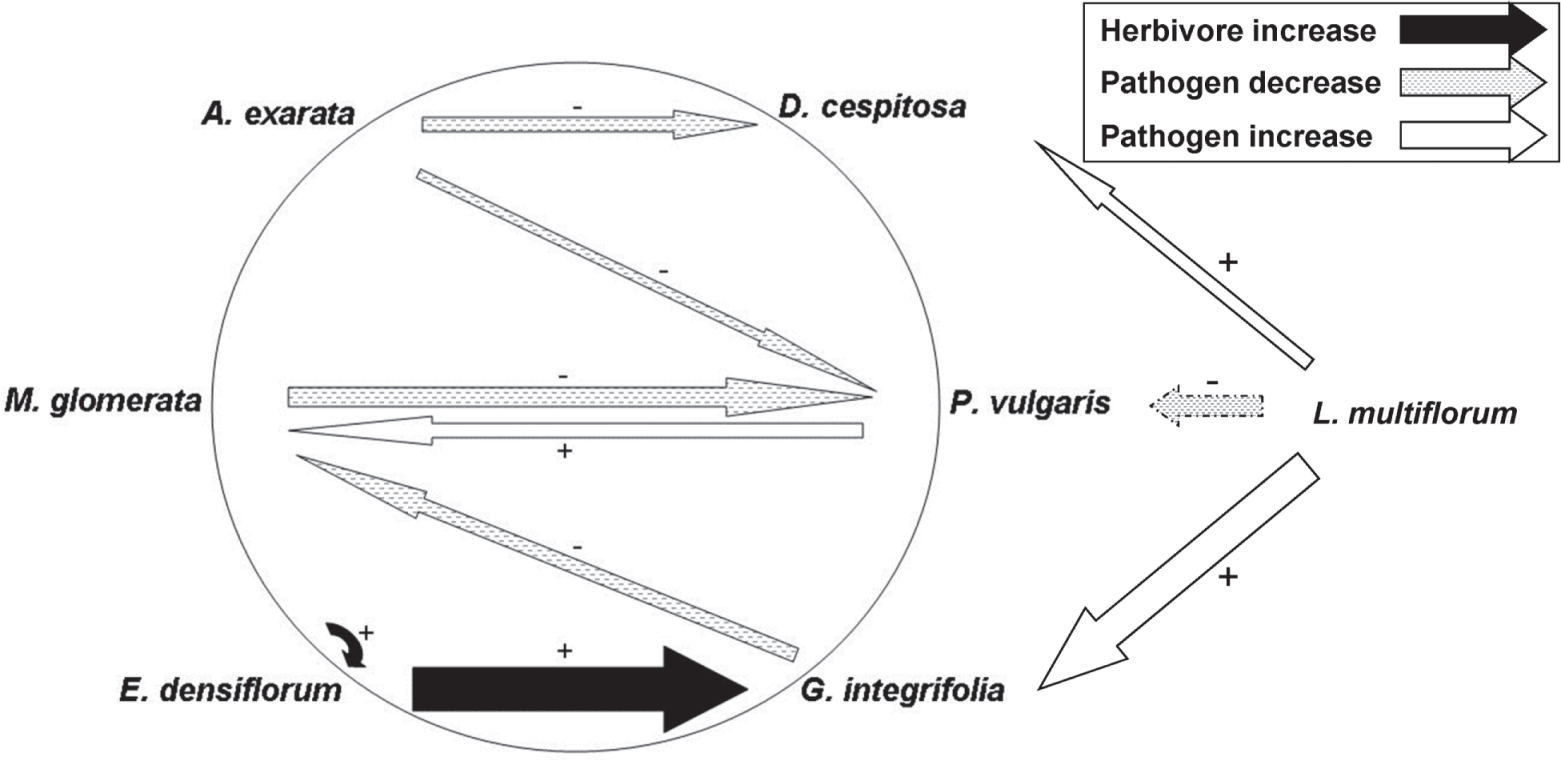
Interpretive Diagram: Partial Correlations of Changes in Herbivore/Pathogen Attack versus Abundance of Plant Species. Diagram is based on the results of our AIC analyses, and represents interactions that were in all or most resulting selected models. Width of arrows indicates approximate magnitude of partial correlation, ranging from 0.06 to 0.40. The arrow with a dashed border was selected in 89% of the models selected. All other relationships shown were selected in 100% of the models.

The correlations of each native plant species’ relative abundance with natural enemy attack on other plant species varied and were plant species-specific (Fig. 3), suggesting that there were no strong community-wide spillover or dilution effects. As *E*. *densiflorum* relative abundance increased, herbivory on itself and on *G*. *integrifolia* also increased, suggesting possible spillover of an herbivore from *E*. *densiflorum* to *G*. *integrifolia*. The most common herbivore damage on both species was caused by chewing insects (Table 2), which indicates that the two host species may share a common herbivore. Consistent with dilution by the two other native species, pathogen attack on *P*. *vulgaris* decreased as relative abundance of *M*. *glomerata* and *A*. *exarata* increased (Fig. 3). The native grass *A*. *exarata* also seemed to have a dilution effect on pathogen attack on *D*. *cespitosa*. Pathogen attack on *M*. *glomerata* increased as *P*. *vulgaris* relative abundance increased, but decreased as *G*. *integrifolia* increased.

Physical attributes of the community had host species-specific correlations with natural enemy attack (Fig. 2E, 2F, 2G). As total plant cover increased, pathogen attack on *E*. *densiflorum* and *M*. *glomerata* increased, and appeared in all models selected (Fig. 2E). Pathogen attack on *G*. *integrifolia* decreased as standing thatch increased, and this variable appeared in all selected models (Fig. 2F). No plant species showed a strong change in enemy attack with respect to ground thatch (Fig. 2G).

We found no support for either the plant vigor hypothesis or the plant stress hypothesis. There were no strong community-wide or individual plant species changes in enemy attack with respect to the individual plant traits shoot biomass (Fig. 2H) or chlorophyll content (Fig. 2I).

## Discussion

Some hypotheses that we explored may be affected by the degree of shared natural enemies among host species. For example, an extreme generalist enemy may not be affected by increased host species diversity or abundance of a particular host [56]. Effects of abundance of a particular host species would be stronger with a specialized natural enemy [57]. An absolute specialist on one host species would not contribute to enemy spillover, but may be more strongly associated with a dilution effect by other species. The native grasses *Agrostis exarata* and *Deschampsia cesptiosa*, as well as other native and introduced grass species, are susceptible to the same isolates of *Alternaria spp*. [58]. The level at which natural enemies are shared among these plant species is otherwise unknown. The goal of our study was to explore the relative predictive strengths of several hypotheses through our survey of patterns of damage by natural enemies, rather than to test patterns of damage by specific herbivores or pathogens. Several types of symptoms were observed (Table 2), which were caused by different guilds of natural enemies. It is therefore most likely that there was variation among the natural enemies with respect to their degree of host specialization.

As is generally true of studies in which natural enemies are observed without direct manipulation of those enemies, the natural enemy community in this study could have affected relative abundance or individual traits of the plant hosts, thus affecting our estimate of natural enemy attack. Enemy exclusion studies can provide additional insight into the hypotheses explored in this study, as well as the ability to address other hypotheses that we could not effectively explore in this study [59,60]. For example, natural enemy spillover can lead to apparent competition between host species [61,62], but experimental manipulation is required to tease out the relative effects of resource competition and apparent competition.

The AIC models used to explore several hypotheses for predicting patterns of herbivore and pathogen attack generally explained more variation in pathogen attack than in herbivory. Interestingly, we did not find any correlation between plant species diversity and herbivore attack. Past studies that have found reduced herbivory with increased host diversity have typically focused on agricultural systems with lower species richness than in our study [9,10]. Lau and others [63] found increased herbivory by specialists, but decreased herbivory by generalists with low host diversity. The relationship between herbivore attack and host species diversity may not be as strong in communities of relatively high host diversity as was the case in our study. Of the variables that we addressed, the only consistent community-wide pattern we found was a small negative correlation between pathogen attack and plant species diversity, similar to the findings of other studies [2,63]. Symptoms caused by one particular pathogen can vary greatly among host species. Therefore the symptoms scored on each host species may be due to different pathogens on each host species or due to one or more shared pathogens, but the negative correlation between host diversity and pathogen attack is suggestive of multiple specialist pathogens. The observed diversity effect on pathogen attack did not appear to be an artifact of the abundance of any one particular plant species in the community. Other recent studies of pathogens and host diversity have also supported this finding. Dizney and Ruedas [64] found that as mammalian diversity in forests declined, the proportion of deer mice infected with sin nombre virus increased. High diversity in ecological communities has also been shown to reduce the frequency of Lyme disease [4], frog malformations caused by parasites [1], and foliar pathogens of plants [2,63]. An effect of host abundance was supported only by an increase in herbivory on *Epilobium densiflorum* as its abundance increased. We found no evidence of a community-wide trend in host abundance and attack on each host species, despite large differences in host abundance among the communities.

Evidence of spillover and dilution effects was also plant species-specific, with no community-wide trends. We found plant species-specific correlations among the relative abundance of one species and attack on another, consistent with enemy spillover and dilution. This result held for both herbivore and pathogen attack, and relative abundances of both native and introduced host species were correlated with increased or decreased enemy attack on another plant species. We also did not find a community-wide trend in spillover from introduced species, as might have been predicted based on Parker et al. [22], or a stronger link between confamilial host species as might have been predicted by Ricciardi et al. [23]. However, our study did not directly test spillover and dilution. To clearly discern spillover and dilution, there is a need for future community-wide studies that identify the herbivores and pathogens on all hosts.

Physical community structure revealed host species-specific correlations, but no community-wide trends in enemy attack. Our method of estimating thatch, in two dimensions only, may have obscured variability at higher thatch densities distributed vertically through the plant canopy, possibly hampering our ability to detect an effect of thatch.

We found no support for the plant vigor or plant stress hypotheses as there were no correlations between individual plant traits and natural enemy attack. As conditions were relatively homogenous throughout our study area, it may be that sufficient variation in plant vigor was lacking to detect an effect. It is also plausible that natural enemies preferentially attacked more vigorous plants and reduced their biomass, which would have obscured our ability to detect evidence of the plant vigor hypothesis.

### Conclusions

Increased host species diversity reduced attack by pathogens, although the effect was not large. This negative correlation is consistent with the findings of numerous other studies across a broad range of host-pathogen systems. Compared to the general negative relationship between host diversity and pathogen attack, effects of other variables, such as relative abundance of individual host species and physical characteristics of the host community, were stronger but varied in magnitude and direction among host species. No single hypothesis provided the ability to predict the strongest trends in natural enemy attack across the host community. The strength of each hypothesis we explored may vary among plant communities and depend largely on the level of specialization of the dominant natural enemies in a particular plant community.

## Supporting Information

S1 File Site Preparation Techniques and Natural Enemy Attack. (PDF)

S2 File Data Used for Analyses Reported in this Study. (XLSX)
