# Supplementary material for: An Exploration of Hypotheses that Explain Herbivore and Pathogen Attack in Restored Plant Communities
Source: PLoS One. 2015 Feb 20;10(2):e0116650. doi: 10.1371/journal.pone.0116650 (PMC4336146; doi:10.1371/journal.pone.0116650)
Supplement: S1 File — (PDF) [file pone.0116650.s001.pdf]

File S1. Supporting Information 1.

Site preparation techniques and natural enemy attack

To compare herbivore and pathogen damage among restoration treatments, we performed two-way ANOVAs for herbivore and pathogen damage, with plant species, restoration treatment or reference site, and species-treatment interactions as independent variables. The interactions among treatment combinations and the response of plant species community composition to these treatments were explored in further detail by Pfeifer-Meister et al. [37]. We considered treatment a fixed variable and species a random variable. Because of inadequate replication of *E. densiflorum* and *G. integrifolia* in two of the treatments, two ANOVAs were run for herbivore and pathogen damage. One included the four remaining species and all treatments, and the other included all six species but excluded the reference and till + solarization treatments (Tables S1, S2, S3, and S4). Post hoc Tukey's HSD tests were performed to determine differences among individual treatments and plant species ($P < 0.05$).

Herbivore attack varied among plant species ($F_{5,40} = 9.03$, $P < 0.0001$) with no main treatment effect; there was an interaction between species and treatment ($F_{48,225} = 2.85$, $P < 0.001$, Figure S1). Herbivore damage was significantly higher on *Grindelia integrifolia* than on the other five species, which did not differ significantly from each other. Herbivore damage was higher in the herbicide treatment than in the till + thermal treatment only when *G. integrifolia* and *E. densiflorum* were included. This difference was driven by high herbivory on *G. integrifolia* in the herbicide treatment. When these rare species were excluded and all treatments were included, herbivory did not vary among treatments and there was no interaction between species and treatment.

Pathogen attack varied among plant species ($F_{5,40} = 19.26$, $P < 0.0001$) but not among the ten restoration treatments and reference wetland prairie (Figure S1). There was more pathogen attack on *Agrostis exarata* than on the other five species. There was an interaction between treatment and species only when *Grindelia integrifolia* and *Epilobium densiflorum* were excluded from the analysis and all treatments were included, because these two species were absent from some treatments ($F_{30,174} = 1.70$, $P = 0.019$); pathogen attack on *Prunella vulgaris* was higher in the reference than in the restoration treatments. Overall, there was not a strong community-wide effect of treatment on herbivore or pathogen attack.

We did not find a strong community-wide effect of site preparation technique on natural enemy attack in the restoration experiment. Our survey of the ten treatments and reference was motivated by dramatic differences in the plant community composition among treatments during the previous growing season, but these differences were less pronounced during the growing season in which this study was conducted [37,38]. Additionally, the plant community of the restoration experiment became more similar to that of the remnant prairie site with respect to plant species composition [37,38]. Therefore it is not surprising that we found few differences in natural enemy attack among the restoration treatments. While we observed minimal effects of the site preparation treatments on enemy attack on native plants, restoration practices that produce larger differences in plant communities may find larger effects.

Figure S1. Herbivore and Pathogen Damage to Six Native Plant Species among Restoration Treatments and Reference Prairie. (mean ± standard error) Different lowercase letters represent significant differences in herbivory among restoration treatments with all six plant species included (Tukey's HSD: $P < 0.05$); when rare plant species were excluded and all treatments were included. There was no significant main effect of treatment. We found no significant main effect of treatment for pathogens, although pathogen damage on *Prunella vulgaris* was higher in the reference than in the restoration treatments.

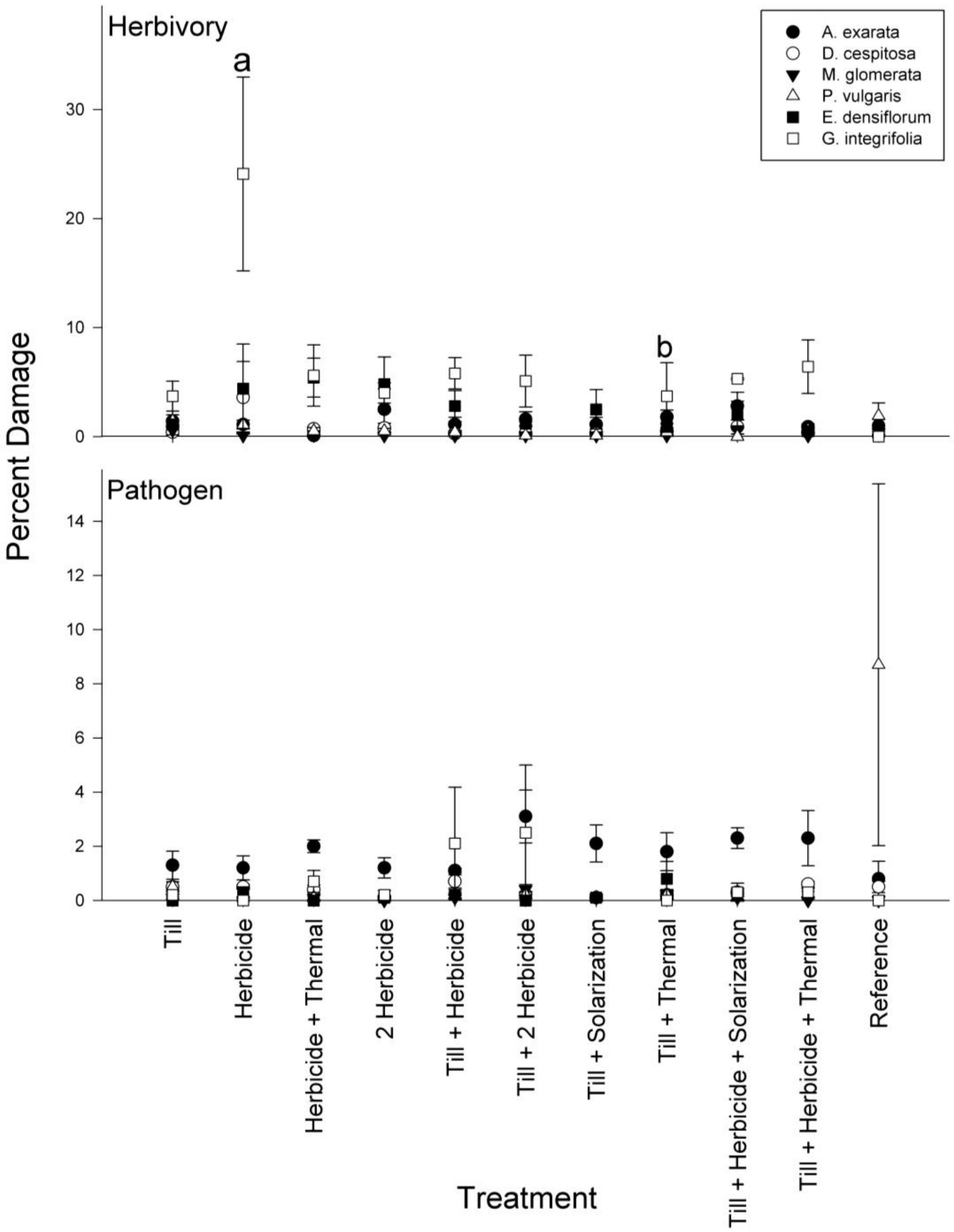
Herbivory
Pathogen
a
b
A. exarata
D. cespitosa
M. glomerata
P. vulgaris
E. densiflorum
G. integrifolia
Percent Damage
0
10
20
30
0
2
4
6
8
10
12
14
Till
Herbicide
Herbicide + Thermal
2 Herbicide
Till + Herbicide
Till + 2 Herbicide
Till + Solarization
Till + Thermal
Till + Herbicide + Solarization
Till + Herbicide + Thermal
Reference
Treatment

Table S1. Herbivory, Two Plant Species Excluded.

Source	SS	MS Num	DF Num	F Ratio	Prob > F
Treatment	24.2762	2.42762	10	1.0456	0.4316
Species	39.11	13.0367	3	5.6147	0.0035*
Treatment x Species	69.6451	2.3215	30	0.8854	0.6412

Table S2. Herbivory, Two Site Treatments Excluded.

Source	SS	MS Num	DF Num	F Ratio	Prob > F
Treatment	48.7588	48.7588	8	2.3551	0.0581
Species	1063.42	212.684	5	0.6025	< 0.0001*
Treatment x Species	951.558	23.7889	40	2.8459	< 0.0001*

Table S3. Pathogen, Two Plant Species Excluded.

Source	SS	MS Num	DF Num	F Ratio	Prob > F
Treatment	65.4518	6.5418	10	0.6805	0.7337
Species	84.164	28.0547	3	2.9170	0.0503
Treatment x Species	288.682	9.62272	30	1.6977	0.0192*

Table S4. Pathogen, Two Site Treatments Excluded.

Source	SS	MS Num	DF Num	F Ratio	Prob > F
Treatment	10.6589	1.33237	8	1.3907	0.2276
Species	91.9626	18.3925	5	19.2647	< 0.0001*
Treatment x Species	38.0954	0.95239	40	0.8572	0.7124
